# Predicting Factors and Clinical Characteristics of Pruritus in Psoriasis: A Cross-Sectional Survey

**DOI:** 10.3390/life14070827

**Published:** 2024-06-28

**Authors:** Silviu-Horia Morariu, Ovidiu Simion Cotoi, Oana Mirela Tiucă, Mircea Ambros, Roxana-Ioana Ilcuș, Liuba Garaga, Ailincăi Raluca, Diana Horea, Andreea-Beatrix Bălan, Mădălina Husariu, Anca Gînj, Alexandra Țiplic, Andrea Hidi, Biborka Szabo, Radu Alexandru Stan, Alin Codruț Nicolescu

**Affiliations:** 1 Dermatology Department, George Emil Palade University of Medicine, Pharmacy, Science, and Technology of Targu Mures, 540142 Targu Mures, Romania; 2 Pathophysiology Department, George Emil Palade University of Medicine, Pharmacy, Science, and Technology of Targu Mures, 540142 Targu Mures, Romania; 3 Dermatology Clinic, Mures Clinical County Hospital, 540342 Targu Mures, Romania; 4 Agrippa Ionescu Emergency Clinical Hospital, 011773 Bucharest, Romania

**Keywords:** psoriasis, pruritus, itch questionnaire

## Abstract

Pruritus is an important symptom among patients affected by psoriasis. To date, no general agreement has been established regarding pruritus as a measure of psoriasis severity. This study aims to assess psoriatic pruritis prevalence and characteristics using a comprehensive itch questionnaire. A semi-structured questionnaire consisting of 48 questions was applied to patients diagnosed with psoriasis and admitted to the Dermatology Department of Mures Clinical County Hospital, Romania. A total of 163 patients were enrolled, out of which 115 (70.55%) reported itch. Patients with itch had higher PASI (*p* = 0.003) and DLQI scores (*p* < 0.001). The itch was most frequently described as a crawling sensation, mainly located in the lesional skin and aggravated by stress and temperature variation. It had a moderate intensity (6.18 ± 2.46). Emollients were the treatment preferred by most patients in alleviating itch, while biologics exerted a protective effect on itch development (OR = −0.24; *p* < 0.0001) and negatively correlated with itch intensity (r = −0.23; *p* < 0.0001). Advanced age, high BMI, and PASI scores were indicators of itch presence, while female gender, high PASI score, and frequent itch episodes indicate highly intense pruritus (≥7 on the VAS). A better understanding of itch and its clinical features will guide physicians toward the best treatment option and would, ultimately, benefit the patient.

## 1. Introduction

Psoriasis is a chronic, immune-mediated disorder affecting up to 3% of the world’s population [1]. It is characterized by a prolonged evolution and frequently associated with an impaired quality of life (QoL) and various comorbidities. Patients with psoriasis are at an increased risk of developing associated metabolic, hepatic, psychiatric, and cardiovascular diseases [2].

Pruritus is considered an unpleasant sensation that triggers the desire to scratch, either consciously or through reflex mechanisms, and affects up to half of the patients suffering from primary dermatologic diseases [3]. Historically, psoriasis was considered an asymptomatic, non-itchy skin disease; moreover, the presence of pruritus was used as a strong discriminator between atopic dermatitis and psoriasis. Four main types of pruritus have been described, namely proprioceptive, systemic, neuropathic, and psychogenic [4]; nevertheless, multiple classifications have been proposed.

Significant advances have been made in recent years regarding psoriasis pathogenesis. An altered immune response in genetically predisposed [5] individuals and response to various triggering factors lead to abnormal keratinocytes hyperproliferation via T helper cell types 1 (Th1) and 17 (Th17) and various cytokines, such as interleukins 17 (IL-17) and 12 (IL-12) [6], resulting in well-defined erythematous lesions covered by thick, silvery scales.

Nevertheless, information regarding psoriasis-associated pruritus has remained sparse. Chemokines, interleukins, mainly Th-17, the P substance, and the nerve growth factor [7] seem to be involved in psoriatic pruritus to a certain extent, indicating the complex, intricate mechanisms that define psoriasis-associated itch. Moreover, no clear answer regarding what triggers pruritus in psoriasis has been given so far, highlighting the need for further research.

A general agreement including pruritus as a measure of quantification of psoriasis severity has not been established so far; however, pruritus prevalence in psoriasis seems to be of interest, with a prevalence varying between 60–90% of cases [8,9,10,11,12]. Despite its relatively high prevalence, the available data regarding the clinical characteristics of itch in psoriasis remain limited, with no clinically comprehensive characterization widely available until now.

This study aims to assess psoriatic pruritis prevalence and its characteristics using a comprehensive itch questionnaire.

## 2. Materials and Methods

### 2.1. Study Design and Population

A semi-structured questionnaire comprising 48 questions was administered in a cross-sectional study conducted between April 2022 and March 2024. The study included patients diagnosed with psoriasis and admitted to the Dermatology Department of Mures Clinical County Hospital, Romania. The questionnaire was distributed to patients with various forms of psoriasis, from mild to severe, who visited the Dermatology Department during the study period and consented to participate. Patients with other dermatological diseases defined by itch or associated comorbidities potentially linked to pruritus, such as renal or hepatic diseases, and pediatric patients were excluded from the analysis.

The primary endpoint of our study was to assess the prevalence of pruritus in psoriasis and its clinical characteristics. The secondary objectives were to identify factors associated with pruritus presence and intensity.

After listening to a brief explanation of the research project and signing the informed consent, patients were invited to complete the questionnaire on itch and psoriasis (for detailed information, please refer to Supplementary Material); completing the questionnaire took approximately 20 min. It was constructed by two dermatologists (S.H.M and O.M.T) and further refined (S.H.M, O.M.T, O.S.C, A.M, R.I.I, and A.C.N). First, we established the questionnaire format, after which each item’s format, content, and length were established. The questionnaire used in this study was designed as a semi-structured one, with both fixed questions, with “yes” and “no” answers, as well as open-labeled questions, in which the patients were asked to describe triggering factors for itch if present, for example. This was carried out in order to obtain both qualitative and quantitative data and to gain a comprehensive view of the characteristics of itch in psoriasis. The patients were asked to complete the questionnaire in paper form and were always assisted during this procedure by a junior dermatologist for possible additional unclarities. If needed, one of the dermatologists who elaborated the questionnaire was also available to address any requests for further details.

Collected data included demographics (age, sex, height, weight, education, and profession), disease presentation (clinical form, lesions’ extension), itch characteristics, worsening and relieving factors, and topical and systemic treatment modalities. Systemic treatment refers to classical agents (methotrexate, cyclosporin, and acitretin), narrow-band UVB phototherapy, and biologics (adalimumab, infliximab, etanercept, ixekizumab, secukinumab, risankizumab, guselkumab, tildrakizulmab, and guselkumab). Previous systemic treatment was checked as a “yes” if the patient had previously undergone or was currently undergoing therapy with any of the aforementioned modalities.

### 2.2. Outcome Measurements

The visual analogue scale (VAS), a 10 cm horizontal line, was used to assess itch intensity, with 0 meaning no itch and 10 indicating a very severe itch. It was graded as follows: absent (0), mild (1–3), moderate (4–6), and severe (7–10). Patients were also asked to indicate the mean number of days the itch was present in the last month before the completion of the questionnaire and to mark, on a structured scale, the moments within a day when the itch was more intense (in the morning, in the afternoon, in the evening, or during the night). The patients were asked to indicate the skin areas affected by pruritus on a schematical representation of the human body inserted into the questionnaire. These data were then correlated with the lesions’ localization.

Disease activity was defined using the PASI score as follows: mild (PASI < 5), moderate (PASI 5–10), and severe (PASI > 10). Psoriatic arthritis association was also investigated; for it to be considered, the patient must have been previously diagnosed by a rheumatologist according to the CASPAR criteria [13].

Life quality was assessed using the Dermatology Life Quality (DLQI) score [14]. It was classified as follows: no (0–1), minimal (1–5), moderate (5–10), important (11–20), and very important (>20) effect on patients’ life quality. Patients were also asked to indicate on the VAS how bothersome the itch was during their daily activities, with 0 being unbothersome, 1–3 being mildly, 4–6 being moderate, and 7–10 being severely bothersome for the patient. Based on pruritus presence, patients were divided into two study groups.

### 2.3. Statistical Analysis

The collected data were anonymized to protect the patients’ confidentiality. The data were then manually introduced into an Excel database (Microsft Office 265, version 2402); in order to limit introduction mistakes, this operation was initially performed by a single person and subsequently checked for accuracy by two other investigators. Statistical analysis was performed using the MedCalc software (version 22.030). Patients’ demographics and disease characteristics were presented as descriptive statistics. The Chi-squared test, with a 95% confidence interval, was used to compare categorical variables, while the Mann–Whitney test or Student’s T-test was used for continuous data. The correlation of different variables was determined using Pearson’s rank correlation. Multiple regression was used to determine factors associated with an itch in psoriasis and a moderate-to-severe itch intensity, when applicable. A level of significance of 0.05 was set.

## 3. Results

### 3.1. Patients’ Characteristics

A total of 267 patients with psoriasis presented to our department, out of which 163 fulfilled the inclusion criteria and were enrolled in this study. A group of 90 men and 73 women, aged from 19 to 87 years old, completed the questionnaire. Most of the patients (n = 145) presented with chronic plaque psoriasis. Joint involvement was noted in 13 patients (7.97%), while 12 patients (7.36%) presented with palmoplantar involvement. The mean disease duration was 17.3 ± 13.55 years, with a maximum of 59 years. The patients’ age at diagnosis ranged from 4 to 75 years.

Of the 163 patients with psoriasis, 115 (70.55%) acknowledged pruritus. Table 1 exemplifies the clinical and sociodemographic characteristics of the patients suffering from psoriasis, with or without the reported existence of pruritus. There were no differences in sociodemographic characteristics between the two study groups. Patients with pruritus presented with significantly higher PASI (*p* = 0.003) and DLQI (*p* < 0.001) scores than those who did not, along with significantly elevated BMI values (*p* = 0.03). No difference was noted regarding the presence of psoriatic arthritis.

### 3.2. Pruritus Characteristics and Daily Activities Impact

Pruritus was frequent in our study group. Most patients described this cutaneous sensation as crawling (n = 94), followed by discomfort (n = 27), burning (n = 23), stinging (n = 19), and pinching (n = 9). Up to 40 patients reported additional associated symptoms such as pain (n = 23) and sweating (n = 17) in the pruritic area.

Itch appeared every other day for most patients (n = 86; 74.78%) and, in most cases, lasted for a few minutes (3.48 ± 1.25). It was located strictly in the affected areas (n = 94; 81.73%) for most patients, with 19 patients reporting it in both lesional and non-lesional skin. It was reported to appear simultaneously in most cases with psoriasis lesions (n = 70; 60.86%) and to have an intermittent character (n = 110; 95.65%).

Patients described pruritus as involving all the areas affected by psoriasis; thus, the most commonly affected areas were the back (74%), legs (62%), arms (45%), and abdomen (38%). The scalp and palmoplantar areas were involved in 6% and 10.4%, respectively.

Most patients acknowledged its presence in the evening (n = 53; 46.08%), followed by the afternoon (n = 27; 23.47%), and during the night (n = 21; 18.26%). Only nine patients (7.82%) indicated that pruritus tended to appear in the morning, while five (4.34%) declared that there was no variation in itch intensity during the day. Up to 60 patients (52.17%) reported that pruritus prevented them from falling asleep easily, while 19 (16.52%) even declared that its existence awakened them.

Most patients acknowledged that pruritus was stronger during stressful events (n = 88; 76.52%); additional triggering factors were reported to be heat (n = 11; 9.56%), cold temperatures (n = 8; 6.96%), sweat (n = 6; 5.22%), or certain foods (n = 5; 4.34%), such as spicy dishes or those containing caffeine. Moreover, pruritus was reported to be more frequent during colder seasons, such as winter (n = 43; 37.39%) and autumn (n = 20; 17.39).

### 3.3. Antipruritic Treatment

Eighty-seven patients (75.65%) admitted that pruritus was influenced by treatment. Itch intensity decreased as topical treatment was applied. Emollients were the most common (n = 40; 34.78%) topical treatment of choice declared to be effective for alleviating itch, followed by corticosteroids alone (n = 10; 8.69%) or in combination with vitamin D3 derivates (n = 3; 2.62%). Applying cold water was also reported to offer a short-term effect on diminishing itch (n = 8; 6,96%). Only two patients underwent systemic treatment for their associated itch, both taking rupatadine when needed.

Our study included 65 patients (39.87%) who received biologics at some point, out of which 33 mentioned pruritus. Treatment duration with biologics was 3.97 ± 2.89 years but with no significant difference in the pruritus vs. non-pruritus group (3.23 ± 2.38 years vs. 4.48 ± 3.20 years; *p* = 0.079). Secukinumab was the most often used (n = 10) in the pruritic group, followed by adalimumab (n = 7), ixekizumab (n = 6), etanercept (n = 5), guselkumab and infliximab (n = 2, each), and risankizumab (n = 1). Significant differences were noted between the patients complaining of pruritus undergoing biologics and those who did not in terms of DLQI scores (*p* = 0.02) and itch intensity (*p* = 0.04).

### 3.4. Pruritus Characteristics and Impact on Daily Activities

Most patients (n = 73; 63.47%) declared that their QoL was predominantly impacted by the aspect of cutaneous lesions and their symptoms, with an additional 18 patients (15.65%) reporting that the itch presence affected them the most. When referring to life domains affected by itch, most patients (n = 70; 60.87%) reported that their social life (including family and friends) was diminished due to the itch presence, while 30 (26.09%) considered that their professional activities were also impacted. The remaining patients declared that their activities were not influenced by this symptom.

Pruritus intensity and its impact on daily activities were assessed using the VAS. The itch was considered moderate (6.18 ± 2.46) and severely impacting daily activities (7.48 ± 3.45). Patients with associated pruritus had significantly higher DLQI scores than those not affected by itch (*p* < 0.0001).

**Figure 1:**
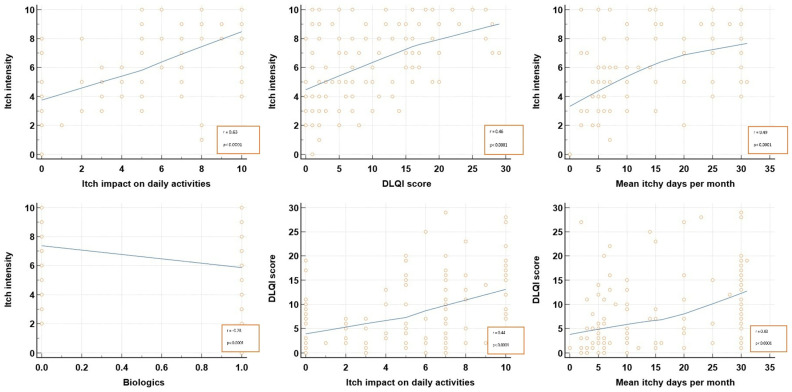
Itch correlation with its characteristics. DLQI, dermatological life quality index.

Pearson’s rank correlation analysis (Table 2; Figure 1) revealed that the DLQI score correlated positively and significantly with itch impact on the daily activities on the VAS as reported by patients (r = 0.435; *p* < 0.0001), as well as with mean itchy days during a month (r = 0.427; *p* < 0.0001). Moreover, itch intensity, as declared by patients, significantly correlated with itch impact on the daily activities on the VAS, the DLQI score, and mean itchy days during a month (*p* < 0.0001).

### 3.5. Factors Predicting Pruritus Presence in Psoriasis

In a multiple regression model (Table 3), older patients (OR = 0.97; >55 years, IQR [52–58] with higher BMI (OR: 1.09; >28.34, IQR [27.34–28.75]) and PASI scores (OR: 1.08; >4.55, IQR [2.90–6.62]) had an increased chance of presenting associated pruritus. Patients who, at some point, underwent treatment with biologics had a significantly lower risk of developing itch (OR: −0.24; *p* = 0.0005). Previous use of classical systemic agents and NB-UVB phototherapy did not impact itch presence.

### 3.6. Factors Predicting Moderate-to-Severe Pruritus in Psoriasis

In a multiple regression model (*p* < 0.0001, Table 4), female patients (OR: 2.63) with higher PASI scores (OR: 1.07) and who frequently experienced itch episodes (OR: 1.12) were most likely to report moderate-to-severe pruritus. Higher PASI scores were defined in our model as above the median value of the pruritus study group of seven (IQR 5.51–9.48).

## 4. Discussion

The prevalence of pruritus in our patients was 70.55%. These data are in accordance with data reported in the literature [8,9,10,11,12,15]. Most patients enrolled in our study had mild psoriasis, both in the pruritic and non-pruritic groups. Both groups mainly included patients diagnosed with plaque psoriasis, with no significant difference in disease subtype. Erythrodermic psoriasis was always associated with pruritus. Moreover, no differences were noted between the two study groups regarding activity status, education level, age at diagnosis, and disease duration, indicating that these factors do not carry weight in reference to pruritus presence in our study groups.

Patients complaining of pruritus had a significantly higher BMI than those in the non-pruritic group. This may be partly explained by the fact that the associated metabolic syndrome can be considered a predisposing factor for itch presence. Lipocalin-2 (LCN2), a protein released by neutrophils and linked to inflammation, insulin resistance, and obesity, is higher in psoriasis than in the controls [16]. Moreover, high serum levels of LCN2 were found to be associated with the degree of itch in patients suffering from psoriasis and decreased after biologic treatment in psoriatic patients complaining of itch.

Patients mentioning pruritus also had a significantly higher median PASI score than those who did not (7 vs. 2.50; *p* = 0.003). This may be because, as the psoriasis advanced, erythema, scaling, and induration, the key components of the PASI score, become aggravated, illustrating a more pronounced inflammatory state. In psoriasis lesions, T cells, dendritic cells, and keratinocytes release various molecules that lead to mast cell degranulation [17], promoting and sustaining itch. On the other hand, severe psoriasis is linked to increased scaling, clinically illustrating skin dryness, manifesting in a structurally altered horny layer, and leading to increased water loss and an auto-irritating mechanism that further promotes neuropeptide release from nerve endings [7].

Pruritus presence was linked in our study to decreased QoL, as measured by both the DLQI score and the VAS. Patients with associated itch had a fivefold lower QoL than those who did not complain of this bothersome symptom. This finding is consistent with those reported by Mrowietz et al. [18], in a post hoc analysis of the Pristine trial, who found that the higher the improvement in itch intensity at 24 weeks of treatment, the bigger the improvement in the patient’s QoL.

Even though itch in psoriasis may vary from mild to severe, most patients reported in the literature describe a moderate intensity of itch (ranging from 4.2 to 6.4 points on the VAS) [8,12,19]. These data are in accordance with the findings of our study, where patients complaining of itch presented with a mean value of 6.18 ± 2.46 on the VAS.

Patients reporting pruritus were diagnosed earlier in life and had a lower disease duration than those who reported psoriasis lesions to be asymptomatic. The findings indicated that the most common description of the associated itch was “crawling” but “discomfort” and “burning” sensations were frequently used as well. The majority of patients felt the pruritus only in their psoriatic lesions. However, a smaller number recollected its presence in both lesional and non-lesional skin (n = 19; 16.52%). Interestingly, two patients complained of itch only on normal-looking skin. This is linked to the fact that non-lesional psoriatic skin, despite not exhibiting clinical signs of disease, lies in an intermediate state between healthy and diseased. Perilesional skin presents with an elevated pH, low-expressed epidermal differentiation, and a propensity for keratinocyte activation and proliferation, as well as IL-17 overexpression [20].

Pruritus tended to appear every other day in most cases and to occur intermittently in the evening. This may be because most patients included in our study were still working or actively involved in various daily activities, and their focus was on completing that specific task. Itch exacerbation may be, in this instance, due to the lack of external stimuli [21] that can lead to rumination and mental stress. The mental load was proved to exacerbate pruritus presence [22]. On the other hand, pruritus exacerbation in the evening may also have a biochemical explanation. In a study published by Wojcik et al. [23], patients with psoriasis presented with elevated levels of eicosanoids, including prostaglandin E1 (PGE1) and thromboxane B2 (TXB2). These molecules, typically released with a circadian pattern, are known to promote inflammation and decrease the cutaneous threshold to itch [24]. As such, we postulate that itch aggravation in psoriasis may be partly due to a disruption in PG and TX circadian rhythms. Future research is, however, needed in this matter.

Even though psoriatic pruritus can affect any anatomical site, itch mostly affects the back, legs, arms, and abdomen. This phenomenon may be partly because these are the areas where psoriasis lesions are normally located and because they are easily reachable by the patient. This may be due to the fact that scratch-induced trauma may lead to the onset of new lesions, namely the Koebner phenomenon [25], which will subsequently lead to increased angiogenesis and inflammation processes [26]. Patients with nail psoriasis are less likely to complain of pruritus, probably due to the clinical manifestations such as onychorrexis, onychogriphosis, and nail crumbling that may make the chronic and vicious itch–scratch cycle difficult.

Stress was commonly reported to exacerbate itch, highlighting once more the complex etiopathogenesis of psoriasis, including the psychosocial part. Stressful events could intensify mast cell degranulation [27] and intensify, via the hypothalamic–pituitary axis, hormonal secretion, mainly the corticotropin-releasing hormone [28]. Other important factors reported to increase pruritus were temperature variations (too hot and too cold) and sweat. These are probably linked to increased inflammation underlying the psoriasis lesions. Another possible explanation is that the hyperhidrosis and increased temperature-associated vasodilation might be linked to increased transepidermal water loss that can initiate the itch–scratch cycle. On the other hand, lower temperatures lead to vasoconstriction, skin xerosis, horny layer, and hydrolipidic film anomalies.

By the current consensus, neither itch presence nor intensity are part of the available armamentarium for quantifying psoriasis severity; moreover, itch management is not part of the currently available guidelines for treating psoriasis. However, we consider it an important and integrative part of the approach to such patients since itch presence and intensity significantly diminish patients’ quality of life. Most patients complaining of itch have mainly used emollients to relieve the itch. Furthermore, steroids have been proven to be useful in alleviating itch, while only two patients mentioned a beneficial effect from oral antihistamines. However, since most of the patients from our study group have relied on emollients to diminish the associated itch, we suggest that one of the main, if not the main, pathogenic mechanisms in psoriatic pruritus is the mechanical one, with skin dryness being the determinant factor. Moreover, mechanical itch can be triggered by simple maneuvers or objects, such as clothing touching lesional skin. Our patients complained of pruritus mainly in cloth-covered areas such as the back, limbs, and abdomen, and not on exposed skin.

Regarding data referring to treatment of choice, the existing data are conflicting. Antihistamines were reported to be effective in alleviating psoriatic itch in a previous study; however, minimal data are available regarding their usefulness in psoriasis [29]. On the other hand, emerging data have indicated that histamine has a limited effect in generating and maintaining itch in psoriasis; alterations in the tropomyosin-receptor kinase A–nerve growth factor axis modulate the aggravation of histamine-independent itch [30]. Phototherapy seems to lead to a decrease in itch intensity [8,12,31] but in our study, previous phototherapy or classical systemic agents do not impact itch presence at intensity. In our study, the previous use of biologics in the group of patients complaining of pruritus leads to lower DLQI scores and itch intensity compared to the subgroup not on biologics. Moreover, biologic use proved to be a protective factor against itch development (OR: −0.24; *p* < 0.01) and negatively correlated with itch intensity (r = −0.23; *p* < 0.0001). However, it was not found to be one of the factors predicting a moderate-to-severe pruritus intensity. Patients undergoing biologics likely experienced intense itching before starting the course of treatment, which then subsided. In a systematic review and meta-analysis published by Therene et al. [32], agents such as etanercept, adalimumab, or ixekizumab significantly improve psoriatic itch. Moreover, it seems that anti-IL-17 agents, such as ixekizumab, lead to a faster and stronger decrease in itch intensity [33,34]. This phenomenon is most likely explained by the fact that IL-17A is highly expressed in the dorsal root ganglion neurons, while, at the same time, stimulating these neural components and enhancing itch signals directly or indirectly [35,36]. Future studies in this matter might prove useful, serving as an important aiding factor for the physician in selecting the best choice of treatment.

In terms of quality of life, both the aspect of the lesions and the associated itch impacted the patients, where the greater the number of itchy days per month and itch intensity, the higher the DLQI scores. A positive significant correlation (r = 0.44; *p* < 0.0001) was noted between life quality, as reported by the calculated DLQI scores, and the patient’s reported itch impact on daily activity on the VAS, indicating that the VAS was a reliable tool in appreciating patient’s life quality.

Regarding factors predicting itch presence, our study identified that advanced age, higher BMI, and PASI scores predispose patients to associated itch. At the same time, previous treatment with biologics, as defined by our methodology, protects patients from developing this symptom. This may be due to the fact that biologics interfere with proinflammatory cytokine production and, in the long run, decrease systemic inflammation and disease activity scores. On the other hand, female patients (OR: 2.63; *p* = 0.04) with higher PASI scores (OR: 1.07; *p* = 0.03) and who frequently reported itch (OR: 1.12; *p* < 0.01) are at a higher risk of complaining of intense pruritus. These results are in accordance with the positive correlation obtained between itch intensity and the mean itchy days per month (r = 0.49; *p* < 0.0001), serving as further clues that the patients’ perception of itch is not only influenced by its intensity but by frequency as well.

The main limitation of this study is that the patients originated from a single tertiary center; future ideas might include expanding the study population to other centers and a dynamic follow-up of patients undergoing various treatments. Moreover, even though the questionnaire was developed and refined by experienced dermatologists, it was not validated; future directions might focus on testing the reliability and validity of this type of survey. In order to limit possible bias in the completion of the questionnaire, it was always completed by patients in the presence of junior dermatologists. In order to gain a comprehensive understanding of itch characteristics in psoriasis, we included patients suffering from various degrees of severity.

## 5. Conclusions

The prevalence of pruritus in psoriasis in our study group was 70.55% and had a moderate intensity, measured on the VAS. Advanced age, higher BMI, and PASI score predisposed patients to presenting itch. When present, the itch appeared daily, mostly in the evening, was located on lesional skin, and significantly affected patients’ quality of life. Emollients were the most effective in alleviating itch. A better understanding of itch and its clinical features and integration into future psoriasis guidelines will guide physicians toward the best treatment of choice. This would, ultimately, benefit the patient.

## Figures and Tables

**Table 1:** Sociodemographic characteristics of the study group.

Characteristics	With Pruritus (n = 115)	Without Pruritus (n = 48)	*p*-Value
Age (years) (mean ± SD)	51.74 ± 16.55	56.93 ± 14.39	0.06
Age at diagnosis	33 (28–38.48)	34 (28.74–40.27)	0.69
Disease duration	13 (10–16.48)	20 (14.74–28.26)	0.15
Sex, n (%)			
Male	54	29	0.06
Female	61	19	
Psoriasis subtype, n (%)			
Plaque	102 (88.69%)	43 (89.58%)	
Pustular	10 (8.70%)	0	0.28
Guttate	1 (0.87%)	5 (10.42%)	
Erythrodermic	2 (1.74%)	0	
PASI score, median (IQR)	7 (5.51–9.48)	2.50 (1.15–4.10)	**0.003**
PASI < 5, n (%)	47 (40.87%)	32 (66.66%)	
5 ≤ PASI ≤ 10,n (%)	30 (26.09%)	8 (16.67%)	0.23
PASI > 10, n (%)	38 (33.04%)	8 (16.67%)	
BMI, median (IQR)	28.41 (27.53–30.24)	27.49 (26.10–28.84)	**0.03**
DLQI, median (IQR)	10 (7–12)	2 (1–3)	**<0.001**
Psoriatic arthritis			
Yes, n (%)	8 (6.96%)	5 (10.42%)	0.06
No, n (%)	107 (93.04%)	43 (89.58%)	
Nail involvement			
Yes, n (%)	5 (4.35%)	13 (27.08%)	**<0.001**
No, n (%)	110 (95.65%)	35 (72.92%)	
Palmoplantar involvement			
Yes, n (%)	10 (8.70%)	10 (20.83%)	**0.03**
No, n (%)	105 (91.30%)	38 (79.17%)	
Activity status			
Retired, n (%)	49 (42.61%)	28 (58.33%)	0.14
Working/student, n (%)	66 (57.39%)	20 (41.67%)	
Education years			
<8, n (%)	21 (18.26%)	5 (10.41%)	
9–12, n (%)	57 (49.56%)	34 (70.84%)	0.19
>12, n (%)	37 (32.18%)	9 (18.75%)	
Previous biologics *			
Yes, n (%)	33 (28.69%)	32 (66.66%)	0.33
No, n (%)	82 (71.31%)	16 (33.34%)	
Previous classical agents *			
Yes, n (%)	52 (45.22%)	21 (43.75%)	**0.01**
No, n (%)	63 (54.78%)	27 (56.25%)	
Previous NB-UVB phototherapy			
Yes, n (%)	60 (52.17%)	16 (33.34%)	0.16
No, n (%)	55 (47.83%)	32 (66.66%)	

PASI, psoriasis area severity index; BMI, body mass index; DLQI, dermatological life quality index; NB-UVB, narrow-band ultraviolet B; SD, standard deviation; IQR, interquartile range; * as defined in the Section 2; statistically significant results (*p* < 0.05) highlighted.

**Table 2:** Correlation between itch and its characteristics.

Marker	r	*p*-Value
Itch intensity		
Itch impact on daily activities	0.63	<0.0001
DLQI score	0.46	<0.0001
Mean itchy days per month	0.49	<0.0001
Biologics	−0.28	<0.0001
DLQI		
Itch impact on daily activities	0.44	<0.0001
Mean itchy days per month	0.43	<0.0001

DLQI, dermatological life quality index.

**Table 3:** Predictors of pruritus presence in psoriasis.

Parameter	OR	95% CI	*p*-Value
Age	0.97	0.94–1.01	0.06
BMI	1.09	1.01–1.17	0.01
PASI	1.08	1.01–1.16	0.02
Biologics	−0.24	−(0.54–0.11)	<0.01

BMI, body mass index; PASI, psoriasis area severity index.

**Table 4:** Predictors of intense pruritus in psoriasis.

Parameter	OR	95% CI	*p*-Value
Female gender	2.63	1.04–6.66	0.04
PASI	1.07	1.01–1.13	0.03
Mean itchy days per month	1.12	1.06–1.17	<0.01

PASI, psoriasis area severity index.

## Data Availability

All data presented can be made available upon reasonable request.

## Supplementary Materials

The following supporting information can be downloaded at: https://www.mdpi.com/article/10.3390/life14070827/s1.
