# Supplementary material for: Predicting Factors and Clinical Characteristics of Pruritus in Psoriasis: A Cross-Sectional Survey
Source: Life (Basel). 2024 Jun 28;14(7):827. doi: 10.3390/life14070827 (PMC11277965; doi:10.3390/life14070827)
Supplement: Supplementary file 1 [file life-14-00827-s001.zip › life-3074195-supplementary.pdf]

Name, surname: □
Phone number: □
Consult date: □
Type of consult: □
Initial visit: □
Check-up visit: □
Patients' photographing: □
Archived data: □
Signed informed consent: □
The patient has been introduced to the psoriasis database: □
Clinical evaluation: □

ITCH EVALUATION QUESTIONNAIRE FOR PATIENTS WITH PSORIASIS:

I. General data :

1. Age .... years
2. Gender ○ M ○ F
3. Residence area
   ○ Urban ○ Rural
4. Educational level (last graduated education form)
   ○ Gymnasium ○ Highschool ○ College ○ Vocational ○ Postgraduate
5. Profession
   Active ○ Unemployed ○ Retired ○ Student

II. Patient's evaluation form:

1. Onset age □□□□
2. First diagnosis □□□□
3. Was a skin biopsy performed?
   ○ a. Yes
   ○ b. No
4. Attached copy ○ Yes ○ No
5. Clinical form (at this examination)
   ○ Guttate ○ Scalp psoriasis
   ○ Vulgar ○ Inveterate psoriasis
   ○ Erythroderma ○ Sebopsoriasis
   ○ Arthropatic psoriasis ○ Nail psoriasis
   ○ Generalized pustular psoriasis
   ○ Pustular palmoplantar psoriasis Hyperkeratotic palmoplantar psoriasis
   ○ Inverse psoriasis
   ○ Mucous psoriasis

6. Clinical form (at the onset of disease)
   ○ The same as the one the patient currently presents with
   ○ Other
   ○ ....
   ○ ....
7. Disease evolution
   ○ Constant
   ○ Dynamic
   ○ The lesions improve or dissapear
   ○ The lesions worsen and spread

Translation from the original version in Romanian.

8. Remission time periods
   - ◯ No
   - ◯ Yes
     - ◯ Number of months..............

9. Topical therapies
   a. Steroids Yes No Currently Name ....
   b. Calcipotriol Yes No Currently Name ....
   c. Steroids+calcipotriol Yes No Currently Name ....
   d. Reductors Yes No Currently Name ....
   e. Keratolytic agents Yes No Currently Name ....
   f. Emollients Yes No Currently Name ....
10. Systemic medication (duration, dose, starting year)
   a. Methotrexate Yes No Currently Name ....
   b. Cyclosporine Yes No Currently Name ....
   c. Retinoids Yes No Currently Name ....
   d. Biologics Yes No Currently Name ....
11. Phototherapy
   - ◯ No
   - ◯ Yes
     - ◯ UVA
     - ◯ UVB
     - ◯ UVB-NB
     - ◯ PUVA
12. Other treatments
   - ◯ No
   - ◯ Yes
13. Comorbidities
   - ◯ Hypertension
   - ◯ Heart failure
   - ◯ Other cardiac diseases..
   - ◯ Diabetes
   - ◯ Dyslipidemia
     - o Mixed
     - o High cholesterol
     - o High tryglicerides
   - ◯ Allergic reactions
     - o Drug-induced
     - o Food-induced
   - ◯ Headaches
   - ◯ Tonsillitis
   - ◯ Urinary tract infections
   - ◯ Asthma
   - ◯ Other pulmonary diseases..
   - ◯ Crohn disease
   - ◯ Ulcerohemoragic rectocollitis
   - ◯ Gastritis
   - ◯ Other digestive diseases...
   - ◯ Surgical interventions...
14. At-home medication for the associated comorbidities:
   - ◯
   - ◯
   - ◯
   - ◯

Translation from the original version in Romanian.

15. Other dermatological diseases
    - ◯
    - ◯
    - ◯
16. Family history of other dermatological and systemic conditions:
    - ◯
    - ◯
    - ◯

III. Symptoms:

1. Does something bother you skin-wise?
   - ◯ Yes
   - ◯ No
2. How would you describe the sensation that you feel cutaneously?
   - ◯ Sting
   - ◯ Discomfort
   - ◯ Crawling
   - ◯ Burn
   - ◯ Pinch
   - ◯ Pain
3. Itch
   - ◯ Present
   - ◯ Absent
4. The itch is located:
   - ◯ Only in areas affected by psoriasis
   - ◯ Only on normally-looking skin
   - ◯ Both
5. The itch started:
   - ◯ Before the onset of the rash
     - o How long before? ....
   - ◯ At the same time as the rash
   - ◯ After the rash
     - o How long after?..................
6. The itch is:
   - ◯ Continuous
   - ◯ Intermittent
7. When is the itch stronger?
   - ◯ In the morning
   - ◯ In the afternoon
   - ◯ In the evening (18:00hr-22:00hr)
   - ◯ At night (>22:00hr)
   - ◯ It is a constant
8. If referred to topical treatment, when is the itch stronger?
   - ◯ Before applying the treatment
   - ◯ After applying the treatment
9. How long does the itch last?
   - ◯ Minutes
   - ◯ Hours
10. During stressful events, the itch:
    - ◯ Is missing
    - ◯ Is stronger
    - ◯ Is milder
11. Do you have difficulties falling asleep due to the itch?
    - ◯ Yes
    - ◯ No

Translation from the original version in Romanian.

12. Has ever happened that you woke up during the night because of the itch?
    - ○ Yes
    - ○ No
13. Which of the following aspects of your life is affected by the itch?
    - ○ Family life
    - ○ Activities with friends
    - ○ Workplace
    - ○ Workplace activity
    - ○ None of the above
    - ○ Other......................................
14. Did you identify any cause of the itch?
    - ○ .
    - ○
15. Your life quality was the most affected by:
    - ○ The skin aspect
    - ○ The associated symptoms (the itch)
16. Percentage-wise, how much of your life quality was impacted by lesions' aspect and how much by the itch?
    - ○ Skin aspect........%
    - ○ Symptoms ....%
17. Is the itch influenced by treatment?
    - ○ No
    - ○ Yes
        - Please mention the type of treatment that relieves your itch:.....................................
18. What do you do when your skin itches?
    - ○
    - ○
    - ○
    - ○
19. Did you ever feel articular pain?
    - ○ No
    - ○ Yes
        - If yes, where?...

IV. Patient's perception: (To be completed by the patient)

1. Please evaluate on the following scale the itch impact on daily activities

0 1 2 3 4 5 6 7 8 9 10

Not bothersome — Severely bothersome

2. Please indicate the average number of days when you felt itch during the last month:

☐

3. Please evaluate on the following numeric scale itch intensity (0=absent; 10=the strongest itch possible)

0 1 2 3 4 5 6 7 8 9 10

Translation fro

4. Please indicate on the following scale the moment during the day when the itch is the most bothersome:

In the morning	In the afternoon	In the evening	At night
☐	☐	☐	☐

5. Please indicate on the following scale the hour when the itch is the most bothersome:

1 2 3 4 5 6 7 8 9 10 11 12 13 14 15 16 17 18 19 20 21 22 23 24

6. In which season is the itch the strongest?
   a. Winter
   b. Spring
   c. Summer
   d. Autumn
   e. It is not seasonally influenced

7. How many minutes does an itch episode last (on average)?

8. Please indicate on the following diagram the areas affected by itch:

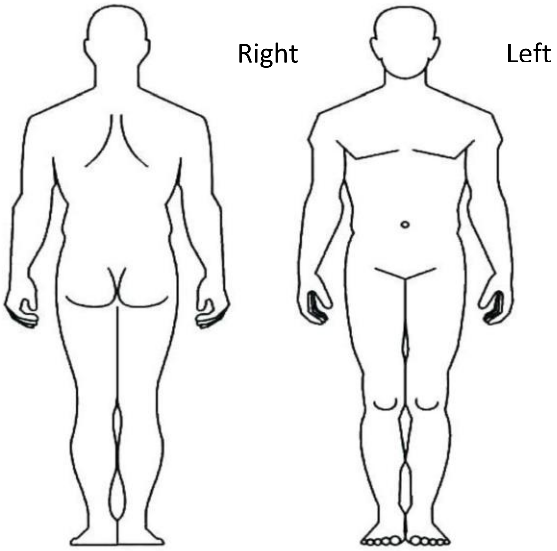

Translation from the original version in Romanian.

9. Please indicate on the following diagram the painful joints:

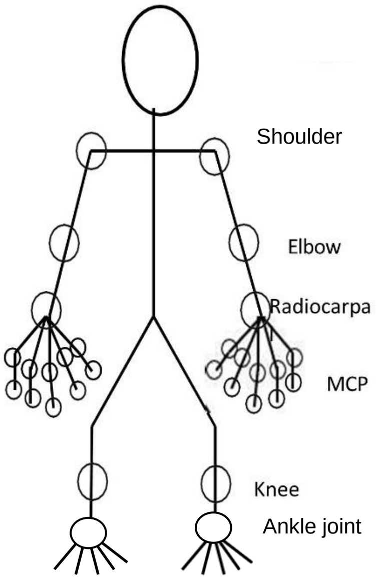

V. Clinical evaluation (To be completed by the physician)

1. Nail involvement
   - ○ Yes
   - ○ No
2. Hair involvement
   - ○ Yes
   - ○ No
3. Please indicate on the following diagram the areas affected by psoriasis in your patient.

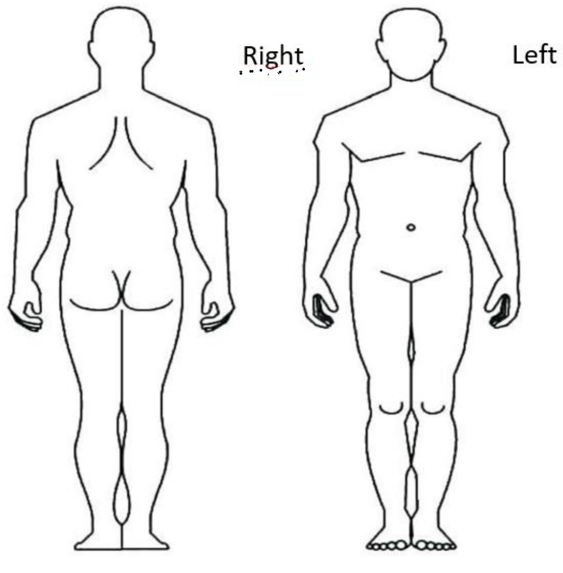

4. Please calculate your patient's PASI score.
5. Please calculate your patient's DLQI score.
6. Please calculate your patient's NAPSIscore.
7. Please calculate your patient's PSSIscore.
8. Please calculate your patient's PGA score.
9. Please calculate your patient's DAPSA28 score.

Translation from the original version in Romanian.
